# Sensitivity and specificity of the Speech, Spatial and Qualities of Hearing Scale (SSQ5) for screening hearing in adults

**DOI:** 10.1590/2317-1782/20212021106

**Published:** 2022-02-28

**Authors:** Rejane Abdala Assef, Katia Almeida, Elisiane Crestani de Miranda-Gonsalez

**Affiliations:** 1 Mestrado Profissional em Saúde da Comunicação Humana, Faculdade de Ciências Médicas da Santa Casa de São Paulo – FCMSCSP - São Paulo (SP), Brasil.

**Keywords:** Questionnaires, Hearing Tests, Sensitivity and Specificity, Hearing Loss, Triage, Questionários, Testes Auditivos, Sensibilidade e Especificidade, Perda Auditiva, Triagem

## Abstract

**Purpose:**

To investigate the sensitivity and specificity of 5 questions of the SSQ in Brazilian Portuguese for its application as a hearing screening instrument in adults.

**Methods:**

A total of 135 adults with a mean age of 49.6 years and education of 9 years took part in the study. All subjects underwent hearing tests and were divided into 2 groups according to hearing acuity: G1 – 66 individuals with normal hearing on audiometric test: and G2 – 69 participants with impaired hearing on audiometric evaluation in one or both ears. The 5 items of the SSQ5, derived from the Brazilian Portuguese version of the SSQ49 were applied. The level of significance was set at a p-value ≤ 0.05, with a 95% confidence interval.

**Results:**

G1 subjects were younger and higher educated (p<0.01). A weak positive correlation was found between education and SSQ5 score only in G1. In G2, there was no correlation of age or education with SSQ5 performance. The area under the ROC curve (AUC) for the relationship between SSQ5 and audiometric average was 0.854 and p-value was <0.001 with bounds of 0.79 and 0.91. SSQ5 scores were lower in G2 (p<0.001). The cut-off point with optimal balance between sensitivity and specificity was 7.3, yielding 80% accuracy, 81.8% sensitivity and 78.3% specificity.

**Conclusion:**

The Brazilian Portuguese version of the SSQ5 proved suitable for screening hearing loss in adults, offering good accuracy, sensitivity and specificity for detecting hearing loss.

## RESUMO

**Objetivo:**

Investigar a sensibilidade e especificidade de cinco questões do SSQ em português brasileiro para sua aplicação como triagem auditiva em adultos.

**Método:**

Participaram 135 adultos com idade de 49,6 anos e escolaridade média de nove anos. Todos foram submetidos à avaliação da audição, e divididos em dois grupos conforme a acuidade auditiva: G1 composto por 66 indivíduos com audiometria normal e o G2 com 69 participantes com audiometria alterada em uma ou em ambas as orelhas. Foram aplicadas as cinco questões do SSQ5 conforme a tradução para o português brasileiro do SSQ49. O nível de significância de p-valor foi definido ≤ 0,05, com intervalo de confiança de 95%.

**Resultados:**

Indivíduos do G1 são mais jovens e escolarizados (p<0,01). Houve correlação positiva de grau fraco entre escolaridade e a pontuação do SSQ5 apenas no G1. No G2 não houve correlação de idade e escolaridade com o desempenho do SSQ5. A área da Curva Roc da relação entre SSQ5 e média audiométrica foi de 0,854 e o p valor foi <0,001 com limites entre 0,79 e 0,91.A pontuação do SSQ5 foi menor no G2 (p<0,001). O ponto de corte do SSQ5 de maior equilibrio entre a sensibilidade e a especificidade foi de 7,3, equivalente a 80% de acurácia, 81,8% de sensibilidade e 78,3% de especificidade.

**Conclusão:**

O SSQ5 em português brasileiro mostrou-se apropriado para triagem da perda auditiva em adultos com boa acurácia, sensibilidade e especificidade na detecção da perda auditiva.

## INTRODUCTION

According to the World Health Organization (2018), handicapping hearing loss affects 432 million adults, one third of whom are over 65 years of age. These estimates are set to rise to 630 million by 2030 and 933 million by 2050. In addition, an estimated 1.1 billion young individuals aged 12-35 years run the risk of hearing loss due to exposure to noise levels in recreational environments.

According to the International Classification of Functioning, Disability and Health – ICF^(1)^, hearing loss stems from problems in function or structures of the auditory system, causing impaired performance of hearing functions, such as: detecting sounds, monitoring the environment, perceiving distance and direction of sounds, locating sound sources and recognizing speech. Any difficulty the individual has performing these tasks characterizes handicap in activities. Disabilities in hearing tasks can handicap the individual in everyday situations, given that these compromise social interaction, relationships, occupational activities, leisure, learning and creativity.

Early detection of hearing loss by a hearing healthcare professional can promote effective interventions. The resources used for hearing rehabilitation reduce communication difficulties by improving hearing performance, thereby allowing greater inclusion and integration of hearing-impaired individuals into society^(2)^. Traditionally, hearing loss is measured by pure-tone threshold audiometry, considered the gold standard for audiologic diagnosis. However, such testing requires a service with the physical infrastructure, equipment and human resources needed for treatment in the case of risk of or suspected hearing impairment.

The ideal strategy for evaluating functional hearing of adults is using a combination of tools, such as questionnaires and audiometric tests^(3-5)^. A variety of different self-assessment instruments have been developed for measuring hearing disabilities, handicap and for documenting self-reported difficulties caused by hearing loss^(6-17)^.

For this purpose, a promising scale is the Speech, Spatial and Qualities of Hearing Scale - SSQ^(18)^ which measures the capacity to listen to speech; localization of sound events for different directions, distances and movement, the listening experience in relation to segregating sounds, identification/recognition, the naturalness and clarity of sounds; musical perception and situations requiring listening effort.

The scale is lengthy and time-consuming to administer, containing 49 items, some of which often go unanswered^(19)^. Consequently, abbreviated versions have been devised with improved clinical applicability and more rapid assessment.

A number of different studies have shown a strong correlation of the original 49-item scale with different versions in other languages, and also with other short forms^(3,5,13,15,17,20-23)^.

The SSQ49 was translated and adapted to Brazilian Portuguese by Gonsalez and Almeida^(22)^. As a follow-up, the same authors carried out a pilot study of a shorter 12-item version (SSQ12) confirming similar mean scores for both the SSQ49 and SSQ12^(24)^.

A study conducted in 2012^(3)^ led to the development of a short SSQ5 version containing only 5 questions to predict the presence or absence of hearing loss in settings where behavioral measurements cannot be carried out. The authors stressed the importance of using measures of disability and handicap to complement measures quantifying hearing loss, such as pure-tone threshold audiometry.

The use of a 5-item scale measuring disability is deemed useful as an instrument for screening hearing loss in adults. However, any scale developed for hearing screening, besides being rapid to apply, should be an accurate method of detecting the presence of impairment, when this indeed exists (sensitivity), while excluding cases without impairment (specificity).

Thus, the purpose of the present study was to investigate the sensitivity and specificity of 5 questions of the short version of the Speech, Spatial and Qualities of Hearing Scale (SSQ5) in Brazilian Portuguese as an instrument for screening hearing in adults.

## METHODS

An exploratory prospective, clinical quantitative study, approved by the Research Ethics Committee of the institution under permit no. 1817374, was conducted. All individuals involved signed the Free and Informed Consent Form. The study involved individuals referred for audiometric testing at a Speech-Language Therapy Clinic.

A total of 135 individuals took part in the present study, selected based on the following eligibility criteria: adults aged >18 years; literate in Portuguese; adequate hearing to understand during interview; and exhibit no apparent cognitive or intellectual deficit.

Participants were divided into two different groups according to the WHO hearing acuity classification^(25)^. Thus, Group 1 (G1) comprised individuals with normal audiometric results (Average _500 1K 2K 4K Hz_≤ 25dBNA) in both ears and Group 2 (G2) with impaired audiometric results (Average _500 1K 2K 4K Hz_> 25dBNA) in one or both ears.

G1 included 66 participants, comprising 30 females and 27 males, with mean age 43.27 (SD 13.22) years, mean education 11.38 (SD 3.61) years, and audiometric average of 8.9 dBNA in the right ear (SD 6.61) and 9.5dBNA in the left ear (SD 7.31). G2 included 69 participants, comprising 51 females and 18 males, with mean age 55.70 (SD 15.66) years, mean education 8.43 (SD 8.43) years, and audiometry average 42.3dBNA in the right ear (SD 26.16) and 45.3dBNA in the left ear (SD 23.90).

All participants were submitted to pure-tone threshold audiometry in a sound-proofed booth using a TDH39 headset. Auditory levels (dBNA) were measured and recorded by air conduction in the 250-8000 Hz frequency range and by bone conduction in the 500-4000 Hz frequency range. The five questions comprising the SSQ5^(3)^, derived from the Portuguese version of the SSQ49 translated by Gonsalez and Almeida^(22)^, were applied (Annex 1). The scale included items involving the 3 core domains: speech-hearing, spatial hearing, and qualities of hearing.

The questions derived from the SSQ49 making up the SSQ5, along with the domains and pragmatic subscales of each question, are shown in Chart 1.

**Chart 1:** Description of domains and pragmatic subscales of the 5 questions derived from the SSQ49 that comprise the SSQ5

**Domains**	**SSQ49**	**SSQ5**	**Item**	**Pragmatic subscale**
Part 1 - Speech-hearing	1.8	1	Can you have a conversation with someone whose voice is the same pitch as that of the person you’re talking with?	Speech in Speech
Part 2 - Spatial hearing	2.3	2	You are sitting in between two people. One of them starts to speak. Can you tell right away whether it is the person on your left or your right, without having to look?	Localization
	2.9	3	Can you tell how far away a bus or a truck is, from the sound?	Distance and Movement
Part 3 - Qualities of Hearing	3.9	4	Do everyday sounds that you can hear easily seem clear to you (not blurred)?	Quality and Naturalness
	3.14	5	Do you have to concentrate very much when listening to someone or something?	Listening Effort

The questionnaire was administered orally in the form of an interview and each response option was rated on a visual analog scale from 0 to 10 points. All subjects marked the condition and situation addressed in each item with a rating, where “0” indicated the individual was unable to perform the hearing function in question (total limitation for activity), whereas “10” indicated ability to perform the activity perfectly (no limitation in activity). In addition, there was an option “not applicable” for cases where the questions did not represent an everyday situation for the interviewee.

Analysis was carried out based on mean total score, which was calculated by summing each item score and dividing by the total number of questions (five). Questions with “not applicable” circled were not included in the calculation of mean total score on the SSQ5.

The level of significance adopted was set at a p-value = 0.005 (5%). Confidence intervals were calculated with a 95% confidence interval. The ANOVA test was applied to determine the SSQ5 scores of G1 and G2. The Receiver Operating Characteristic (ROC) curve was employed to determine the optimal cut-off point on the SSQ and identify individuals from each group showing sensitivity (“patients” – positive on the screening test) and specificity (“non-patients” – negative on the screening test). Pearson's correlation was adopted to measure the degree of correlation between age, education and mean total score on the SSQ5 (SSQ5T) in each group. The Equality of Two Proportions test was adopted to calculate the measures for the relationship between the audiometric four-tone average^(25)^ and the mean total score on the SSQ5 (SSQ5T).

## RESULTS

Descriptive statistics for age and education of the sample of GI and G2 (normal and impaired hearing audiometry groups, respectively), are given in Table 1.

**Table 1:** Descriptive statistics for age (years) and education (years of study) in groups with normal hearing audiometry (G1) and impaired hearing audiometry (G2) (Pearson's Correlation)

**Variable**	**Group**	**N**	**Mean**	**Median**	**SD**	**Min.**	**Max.**	**CI**	**p-value**
Age	G1	66	43.2	43	13.2	18	71	3.19	<0.01
	G2	69	55.7	55.5	15.6	18	89	3.70	
	Total	135	49.6	50	15.7	18	89	2.66	
Education	G1	66	11.3	11	3.6	2	17.5	0.87	<0.01
	G2	69	8.4	8	5.4	2	24	1.33	
	Total	135	9.8	11	4.9	18	24	0.84	

Caption: SD = standard deviation; Min. = minimum score; Max.= maximum score; CI = confidence interval; p-value = significance value

Individuals in the normal hearing audiometry group (G1) were younger and higher-educated than subjects in the hearing-impaired audiometry group (G2). Given the groups differed for age and education (Table 1), correlation of these variables with total score on the SSQ5 was analyzed (Table 2).

**Table 2:** Correlation of Age, Education and total SSQ5 score in group with normal hearing audiometry (G1) and group with impaired hearing audiometry (G2) (Pearson's Correlation)

				**Age**	**Education**
G1	*SSQ*5		Corr (r)	2.4%	24.4%
			*p-value*	0.84	0.04
G2		*SSQ*5	Corr (r)	9.2%	16.5%
			*p-value*	0.45	0.17

Caption: SSQ5 = mean of the five questions; Corr(r) = Correlation; p-value = significance value

The results of this analysis revealed a weak positive correlation between education and mean score on the SSQ5 only in G1, i.e. among the normal-hearing individuals. This relationship indicated that the higher the educational level, the greater the score on the SSQ5. By contrast, there was no correlation of age or education with SSQ5 performance among the hearing-impaired individuals (G2).

The mean score for each of the five questions and mean total score on the SSQ5 (SSQ5T) for G1 and G2 are given in Table 3.

**Table 3:** Descriptive statistical analysis of mean score on SSQ5 and each item, for group with normal hearing audiometry (G1) and group with impaired hearing audiometry (G2) (ANOVA)

**Question**	**Group**	**N**	**Mean**	**Median**	**SD**	**CV**	**Min.**	**Max.**	**CI**	***p-value***
1	G1	66	7.82	8	1.82	23%	3	10	0.44	<0.001
	G2	69	5.39	5	2.29	42%	0	10	0.54	
2	G1	66	8.52	9	1.74	20%	2	10	0.42	<0.001
	G2	69	6.04	6	2.64	44%	0	10	0.62	
3	G1	66	7.65	8	2.00	26%	2	10	0.48	<0.001
	G2	69	5.30	6	2.75	52%	0	10	0.65	
4	G1	66	8.56	9	1.44	17%	5	10	0.35	<0.001
	G2	69	5.94	6	2.28	38%	0	10	0.54	
5	G1	66	7.68	8	2.35	31%	0	10	0.57	<0.001
	G2	69	5.12	5	2.76	54%	0	10	0.65	
Total	G1	66	8.05	8.3	1.32	16%	4.2	10	0.32	<0.001
	G2	69	5.56	5.4	1.94	35%	0.8	9.2	0.46	

Caption: CV = coefficient of variance; Min. = minimum score; Max.= maximum score; CI = confidence interval; p-value = significance value

**Figure 1:**
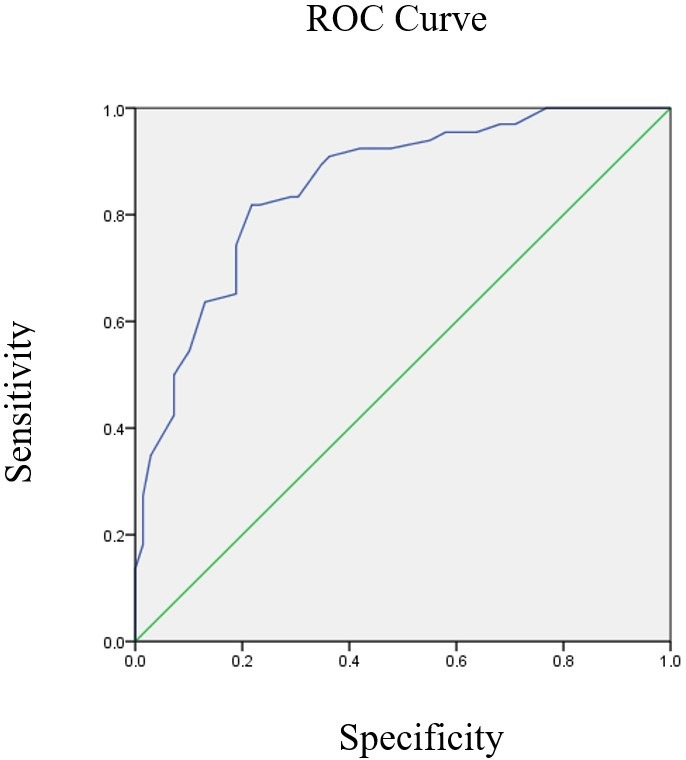
ROC curve depicting mean SSQ5 score and four-tone audiometric average

The ROC curve was plotted for the relationship between mean score on the SSQ5 and audiometric four-tone classification (Average _500 1K 2K 4K Hz_) for all possible cut-off points between 0 and 1. The vertical axis of the ROC curve denotes sensitivity, whereas the horizontal axis represents specificity (Figure 1).

Note that the line obtained (blue) is located toward the top left of the graph and distal relative to the diagonal line (green), indicating a significant AUC value. The AUC for the relationship between mean total SSQ5 and audiometric four-tone classification (Average _500 1K 2K 4K Hz_) was 0.854 with p-value <0.001, lower bound of 0.791 and upper bound of 0.917.

The cut-off point for the SSQ5 providing the optimal balance between sensitivity and specificity for hearing screening diagnosis was 7.3 (Table 4).

**Table 4:** Sensitivity and Specificity of SSQ5 for screening hearing

**Mean SSQ5**	**Sensitivity**	**Specificity**
-0.2	100.0%	0.0%
1.1	100.0%	1.4%
1.7	100.0%	2.9%
2.1	100.0%	4.3%
2.5	100.0%	5.8%
3.1	100.0%	11.6%
3.5	100.0%	14.5%
3.7	100.0%	15.9%
3.9	100.0%	20.3%
4.1	100.0%	23.2%
4.3	98.5%	26.1%
4.5	97.0%	29.0%
4.7	97.0%	31.9%
4.9	95.5%	36.2%
5.1	95.5%	42.0%
5.3	93.9%	44.9%
5.5	92.4%	52.2%
5.8	92.4%	56.5%
6.1	92.4%	58.0%
6.3	90.9%	63.8%
6.5	89.4%	65.2%
6.7	83.3%	69.6%
6.9	83.3%	71.0%
7.1	81.8%	76.8%
**7.3**	**81.8%**	**78.3%**
7.5	74.2%	81.2%
7.7	65.2%	81.2%
7.9	63.6%	87.0%
8.1	54.5%	89.9%
8.3	50.0%	92.8%
8.5	42.4%	92.8%
8.7	34.8%	97.1%
8.9	27.3%	98.6%
9.1	18.2%	98.6%
9.3	13.6%	100.0%
9.5	9.1%	100.0%
9.7	7.6%	100.0%
9.9	4.5%	100.0%
11.0	0.0%	100.0%

After adjusting and determination of the cut-off point, the discriminatory power

of the model was assessed. This assessment entailed calculation of the metrics of Accuracy, Sensitivity, Specificity, Positive Predictive Value (PPV) and Negative Predictive Value (NPV) (Table 5).

**Table 5:** Statistical data for Accuracy, Sensitivity, Specificity and Positive and Negative Predictive Values

**Statistics**	
Accuracy	80.0%
Sensitivity	81.8%
Specificity	78.3%
Positive Predictive Values	78.3%
Negative Predictive Values	81.8%

Comparing the two measurements of tone threshold audiometry and SSG5 score, a positive result on the SSQ5 screening test (< 7.3) was associated with a 78.8% likelihood of hearing loss on audiometry (PPV). Conversely, a negative result on the SSQ% screening assessment (>7.3) correlated with an 81.8% likelihood of normal hearing on audiometry (NPV).

## DISCUSSION

The need for performing hearing tests in the pediatric population is clear, given the impacts of hearing loss, particularly in the development of speech and oral language. However, the same does not seem to hold for the adult population.

In Brazil, 57.1% of the population is aged over 30, while the proportion of individuals aged 65 years or older represents 10.5% of the population (IBGE, 2018) https://educa.ibge.gov.br/jovens/conheca-o-brasil/populacao/18318-piramide-etaria.html. Damage and loss of cilia cells of the inner ear due to aging, exposure to occupational or leisure-related noise, use of ototoxic drugs, chemical products present in the environment and genetic diseases are factors contributing to an increase in the prevalence of hearing loss among the adult population.

Listeners use there auditory system to carry out everyday activities such as listening, hearing, understanding and communicating. Whenever there is a hearing deficit that affects understanding and communications, this negatively impacts the functioning of the individual in dimensions of activities and involvement in activities of daily living.

Therefore, early detection of hearing loss in adulthood is fundamental to avoid the communication and psychosocial losses caused by impaired hearing that impact quality of life. This scenario justifies hearing screening in adults.

Traditionally, hearing tests are done using calibrated equipment which requires sound-proofed testing booths.

However, self-administered scales serve as a good marker of hearing loss in adults, besides providing a more accurate guide for the individual on the need for further diagnostic assessment^(26,27)^. Other notable aspects inherent to the application of these tests include the fact that they dispense with the need for a room with an acoustically controlled environment or specialized equipment and personnel, since these tests do not require the conditions needed for performing audiometry.

The SSQ5 scale yields information on real-life hearing abilities and predicts the presence or absence of hearing loss based on disabilities and handicap in activities of daily living.

In the present study, normal-hearing individuals on audiometry were younger and had higher educational level than hearing-impaired participants (Table 1). However, these variables had no influence on responses to the SSQ5 among hearing-impaired individuals (Table 2).

Literature findings for the SSQ consistent with the normative reference of the present study were compiled to categorize individuals as having clinically-normal or impaired hearing on audiometry.

The different versions applied in previous studies have mean total SSQ scores in the 6.7-8.8 range for normal-hearing subjects and in the 4.1-7.7 range for hearing-impaired individuals, values corroborated by the present study findings^(12,13,15-18,20,22,24,28)^. The SSQ5 used in the present study yielded an average SSQ score of 8.3 points for the normal-hearing participants and 5.4 in the hearing-impaired group. A significant difference between the groups was found for total score on the SSQ5 and for each item (Table 3).

The ROC curve was used to establish the sensitivity, specificity and cut-off point of the SSQ5 (Figure 1) and the presence/absence of hearing loss determined by four-tone average on audiometry^(25)^. The AUC of 0.85 was significant and close to optimal. Values of between 0.8 and 0.9 for interpretation of the AUC in terms of statistical efficiency are considered good indicators of diagnostic quality. A similar study reported less robust areas for the five items of the SSQ (AUC 0.69; p = 0.036) and for the generic question: “Do you have a hearing loss?” (AUC 0.56)^(3)^.

Self-reporting scales assessing disabilities and handicap for activities have been applied as hearing screening instruments in adults and shown higher sensitivity and specificity values than for informal interviews, as in the present study^(3,4)^.

In the literature, specificity and sensitivity percentages for audiological diagnosis for other self-administered scales are available. There is disagreement among some authors on choice of ideal cut-off point in terms of level of sensitivity and specificity. Some authors favor sensitivity for detecting possible hearing impairments, while others prefer specificity for selecting possible cases of normal hearing. Values reported in the literature for the HHIE-S, a scale widely used for screening hearing of older adults, range from 23.5% to 100% for sensitivity and 50% to 95% for specificity^(4,8,9,11)^, whereas cut-offs established for the scale in the present study were 81.8% sensitivity and 78.3% specificity.

In the present study, mean total SSQ5 score was calculated and a cut-off value of 7.3 chosen as the optimal balance point between sensitivity and specificity. According to this cut-off, the hearing of individuals scoring >7.3 on the SSQ5 may be within normal limits. i.e. > 25dBNA in four-tone average^(25)^, whereas subjects scoring <7.3 points may have unilateral or bilateral hearing loss (Table 4).

Analyzing the accuracy of a screening instrument is paramount, providing a measure of the instrument's preciseness and accuracy of the data and information, with absence of errors or mistakes. The Brazilian Portuguese version of the SSQ provides 80% accuracy, corresponding to 80% precision in diagnosing hearing loss for the established cut-off point. This figure indicates greater accuracy compared to other self-assessment hearing handicap questionnaires, which offer accuracy of between 58% and 71.8%^(4,8,9,11,29)^.

The qualitative variables, hearing loss and cut-off point of the SSQ5, besides accuracy, also provide predictive values. Comparing the two measures, audiometry (Average _500 1K 2K 4K Hz_) and SSQ5 cut-off point, a positive result on SSQ5 screening (< 7.3) correlated with a 78.18% likelihood of hearing loss on audiometry testing (PPV). Conversely, testing negative on SSQ5 screening (>7.3) was associated with an 81.8% likelihood of normal hearing on audiometry evaluation (NPV)(Table 5).

In the current study, application of the 5 questions from the SSQ took 3 minutes on average. As expected, the scale containing fewer items had a much shorter test completion time, favoring its application as a hearing screening instrument.

The Brazilian Portuguese version of the reduced 5-item SSQ scale provided rapid assessment with straight forward questions and good specificity and sensitivity for detecting hearing loss. These results suggest its potential for use in hearing screening, both in primary care and epidemiological research settings. This Brazilian Portuguese questionnaire has utility for identifying hearing difficulties and providing more effective referral of adults for diagnostic evaluation.

Further studies of the reduced SSQ5 version, involving larger samples and different settings, should be conducted to elucidate the influence of hearing and non-hearing related variables on scale responses.

## CONCLUSIONS

The Speech, Spatial and Qualities of Hearing Scale in Brazilian Portuguese, version of the reduced 5-item (SSQ5), proved a suitable instrument for screening hearing, providing good accuracy, sensitivity and specificity for detecting hearing loss in adults.

## Supplementary Material

Supplementary material accompanies this paper.

Annex 1 Speech Spatial Qualities Screen (SSQ5) em Português

This material is available as part of the online article from https://www.scielo.br/j/codas

## References

[1] WHO: World Health Organization (2001). International Classification of Functioning, Disability and Health – ICF.

[2] Sousa MGC, Russo ICP (2009). Audição e percepção da perda de audição em idosos. Rev Soc Bras Fonoaudiol.

[3] Demeester K, Topsakal V, Hendrickx JJ, Fransen E, Van Laer L, Van Camp G (2012). Hearing disability measured by the speech, spatial, and qualities of hearing scale in clinically normal-hearing and hearing-impaired middle aged persons, and disability screening by means of a reduced SSQ (the SSQ5). Ear Hear.

[4] Becerril-Ramírez PB, González-Sánchez DF, Gómez-García A, Figueroa-Moreno R, Bravo-Escobar GA, García de la Cruz MA (2013). Hearing loss screening tests for adults. Acta Otorrinolaringol Esp.

[5] Mertens G, Punte AK, Van de Heyning P (2013). Self-assessment of hearing disabilities in cocNBear implant users using the SSQ and the reduced SSQ5 version. Otol Neurotol.

[6] Valete-Rosalino C, Rozenfeld S (2005). Triagem de audição em idosos: comparação entre autorelato e audiometria. Rev Bras Otorrinolaringol.

[7] Noble W, Gatehouse S (2009). Interaural asymmetry of hearing loss, speech, space and qualities of hearing (SSQ) and hearing impairment and performance. Rev Intl Audiolog..

[8] Calviti KCFK, Pereira LD (2009). Sensibilidade, especificidade e valores preditivos da queixa de audição comparados com diferentes médias audiométricas. Rev Bras Otorrinolaringol.

[9] Rosis ACA, Souza MRF, Iorio MCM (2009). Hearing Handicap Inventory for the Elderly -Screening version (HHIE-S): Estudo de Sensibilidade e Especificidade. Rev Soc Bras Fonoaudiol.

[10] Singh G, Kat NB, Pichora-Fuller M (2010). Older adults performance on the speech, spatial, and qualities of hearing scale (SSQ): test-retest reliability and a comparison of interview and self-administration methods. Int J Audiol.

[11] Menegotto IH, Soldera CLC, Anderle P, Anhaia TC (2011). Correlation between hearing loss and the outcome of the hearing handicap inventory questionnaires for the adults-version HHIA-S and screening hearing handicap inventory for the elderly-screening - HHIE-S version. Int Arch Otorhinolaryngol.

[12] Banh J, Singh KG, Pichora-Fuller M (2012). The speech, speech and spatial qualities of hearing range (SSQ) for adults with minimal loss of audiometric. J Am Acad Audiol.

[13] Noble W, Naylor G, Bhullar N, Akeroyd MA (2012). Hearing capabilities self-assessed in adults of ages and larger: a stratified sampling approach. Int J Audiol.

[14] Akeroyd MA, Guy FH, Harrison DL, Suller SL (2014). A factor analysis of the speech, spatial and qualities of hearing scale (SSQ). Int J Audiol.

[15] Mertens G, Hofkens A, Punte AK, De Bodt M, Van de Heyning P (2015). Hearing performance in single-sided deaf cocNBear implant users after upgrade to a single-unit speech processor. Otol Neurotol.

[16] Lotfi Y, Nazeri AR, Asgari A, Moosavi A, Bakhshi E (2016). Iranian version of speech, spatial, and qualities of hearing scale: a psychometric study. Acta Med Iran.

[17] Moulin A, Richard C (2016). Sources of variability of speech, spatial, and qualities of hearing scale (SSQ) scores in normal-hearing and hearing-impaired populations. Int J Audiol.

[18] Gatehouse S, Noble W (2004). The Speech, Spatial and Qualities of Hearing Scale (SSQ). Int J Audiol.

[19] Moulin A, Vergne J, Gallego S, Micheyl C (2019). A new speech, spatial, and qualities of hearing scale short-form: factor, cluster, and comparative analyses. Ear Hear.

[20] Moulin A, Richard C (2016). Validation of a French-Language version of the spatial hearing questionnaire, Cluster analysis and comparison with the speech, spatial, and qualities of hearing scale. Ear Hear.

[21] Noble W, Jensen NS, Naylor G, Bhullar N, Akeroyd MA (2013). A short form of speech, spatial and qualities of hearing scale suitable for clinical use: SSQ12. Int J Audiol.

[22] Gonsalez ECM, Almeida K (2015). Adaptação cultural do questionário SSQ para o português brasileiro. Audiol Commun Res.

[23] Mertens G, Desmet J, De Bodt M, Van de Heyning P (2016). Prospective case-controlled sound localisation study after cocNBear implantation in adults with single-sided deafness and ipsilateral tinnitus. Clin Otolaryngol.

[24] Miranda-Gonsalez EC, Almeida K (2017). Limitação de atividade de audição medida por meio do SSQ Estudo piloto da versão reduzida em português brasileiro. Audiol Commun Res.

[25] WHO: World Health Organization (2014). Prevention of blindness and deafness: grades of hearing impairment..

[26] Kiely KM, Gopinath B, Mitchell P, Browning CJ, Anstey KJ (2012). Evaluating a dichotomized measure of self-reported hearing loss against gold standard audiometry: prevalence estimates and age bias in a pooled national data set. J Aging Health.

[27] Spankovich C, Gonzalez VB, Su D, Bishop CE (2018). Self reported hearing difficulty, tinnitus, and normal audiometric thresholds, the National Health and Nutrition Examination Survey 1999-2002. Hear Res.

[28] Aguiar RGR, Almeida K, Miranda-Gonsalez EC (2019). Test-Retest Reliability of the Speech, Spatial and Qualities of Hearing Scale (SSQ) in Brazilian Portuguese. Int Arch Otorhinolaryngol.

[29] Hashimoto H, Nomura K, Yano E (2004). O estado psicossomático afeta a relação entre as dificuldades de audição subjetivas e os resultados da audiometria. J Clin Epidemiol.

