# Supplementary material for: Sensitivity and specificity of the Speech, Spatial and Qualities of Hearing Scale (SSQ5) for screening hearing in adults
Source: Codas. 2022 Feb 28;34(4):e20210106. doi: 10.1590/2317-1782/20212021106 (PMC9886129; doi:10.1590/2317-1782/20212021106)
Supplement: Annex 1 [file codas-34-4-e20210106-supplementary1.pdf]

Annex 1 - Speech Spatial Qualities Screen (SSQ5) em Português

Speech Spatial Qualities Screen (SSQ5) em Português

Assef, Miranda-Gonsalez (2018)

Orientações sobre como responder as perguntas

As seguintes questões abordam aspectos da sua capacidade e experiência de ouvir e escutar em diferentes situações.

Para cada questão, assinale um (X), em qualquer local da escala apresentada que varia de 0 a 10. Marcar um (X) no 10 significa que você seria perfeitamente capaz de fazer ou experimentar o que está descrito na questão. Marcar um (X) no 0 significa que você seria incapaz de fazer ou experimentar o que está descrito.

Como exemplo, a questão 1 pergunta sobre ter uma conversa com poderia acompanhar cerca de metade da conversa nessa situação deve marcar no ponto médio da escala, e assim por diante.

Esperamos que todas as questões sejam relevantes à sua experiência cotidiana, mas se uma questão descreve uma situação que não se aplica a você, assinale um (X) em "não se aplica". Por favor, também escreva uma observação ao lado da questão explicando porque isso não se aplica ao seu caso.

Por favor, responda às seguintes questões, e então prossiga com o questionário sobre a sua audição.

Nome:

Idade:

Escolaridade (anos):

Assinale uma das seguintes opções:

- ☐ Eu **não** tenho próteses auditivas
- ☐ Eu uso **uma** prótese auditiva (OE)
- ☐ Eu uso **uma** prótese auditiva (OD)
- ☐ Eu uso **duas** próteses auditivas (ambas as orelhas)

Se você usa próteses auditivas, há quanto tempo faz isso?

______ anos ou ______ meses ou ______ semanas

(Parte 1: Audição para a fala)

1. Você consegue conversar com alguém quando há outra pessoa falando e que tem o mesmo tom de voz da pessoa que conversa com você?	De modo algum ... Perfeitamente<br>0 1 2 3 4 5 6 7 8 9 10<br>Não Aplicável ☐
**(Parte 2: Audição espacial)**	
2. Você está sentado entre duas pessoas. Uma delas começa a falar. Você consegue dizer imediatamente se é a pessoa da sua direita ou a da sua esquerda que está falando, sem precisar olhar?	De modo algum ... Perfeitamente<br>0 1 2 3 4 5 6 7 8 9 10<br>Não Aplicável ☐
3. Você consegue dizer o quanto um ônibus ou um caminhão está longe, a partir do seu som?	De modo algum ... Perfeitamente<br>0 1 2 3 4 5 6 7 8 9 10<br>Não Aplicável ☐
**(Parte 3: Qualidades da audição)**	
4. Os sons do dia a dia que você consegue ouvir com facilidade são claros (não turvos)?	De modo algum ... Perfeitamente<br>0 1 2 3 4 5 6 7 8 9 10<br>Não Aplicável ☐
5. Você tem que se concentrar muito quando está escutando alguém ou alguma coisa?	Precisa se concentrar muito ... Não precisa se concentrar<br>0 1 2 3 4 5 6 7 8 9 10<br>Não Aplicável ☐
